# Orthopaedic Fractures among Patients Attending a Tertiary Care Centre

**DOI:** 10.31729/jnma.8325

**Published:** 2023-11-30

**Authors:** Pramod Joshi, Mahesh Karmacharya, Sailendra Kumar Duwal Shrestha

**Affiliations:** 1 Department of Orthopedics, National Trauma Center, Kathmandu, Nepal; 2 Department of Orthopedics, Nepal Armed Police Force Hospital, Kathmandu, Nepal

**Keywords:** *hone fracture*, *orthopedics*, *prevalence*, *tertiary care centre*

## Abstract

**Introduction:**

Orthopedic fractures caused by chronic metabolic bone disease, overuse, or road traffic accidents are among the most significant burdens on society. Furthermore, a growing number of people undergoing arthroplasty and an increase in life expectancy seem to contribute to an increase in orthopaedic fractures. However, research on orthopaedic fracture prevalence or types has been scarce in low- and middle-income countries, including Nepal. The objective of this study was to find out the prevalence of orthopaedic fractures among patients attending a tertiary care centre.

**Methods:**

A descriptive cross-sectional study among patients attending a tertiary care centre was conducted between 19 May and 18 November 2023 during which date from 1 January to 30 December 2021 were collected from the hospital records. Ethical approval was obtained from the Institutional Review Committee. A convenience sampling method was used. Point estimate was calculated at a 95% Confidence Interval.

**Results:**

Among 7609 people, 2518 (33.09%) (12.97-10.55, 95% Confidence Interval) had orthopaedic fractures. A total of 1925 (76.45%) were males. There were 339 (95.49%) fractures associated with two-wheelers and 307 (86.48%) with four-wheelers. There were 1387 (55.08%) soft tissue injuries, 198 (7.86%) skull injuries, and 116 (4.61%) facial injuries. Additionally, there were 73 (2.90%) fractures of the femur, 71 (2.82%) fractures of the phalanx, and 70 (2.78%) fractures of the clavicle.

**Conclusions:**

The prevalence of orthopaedic fractures was found to be higher than in other studies done in similar settings.

## INTRODUCTION

Fractures are breaks in the continuity of a bone that, if not treated properly and promptly, can damage blood vessels and nerves, and infect the bone (osteomyelitis).^1,2^ They may be treated with splints, braces, plaster casts, traction, or even surgically implanted metal plates or rods based on the location and severity of the fracture.^2^

It is estimated that 8.9 million orthopaedic fractures occur each year around the world, including 1.6 million hip fractures, 1.7 million forearm fractures, and 1.4 million vertebral fractures.^3^ One in six deaths from orthopaedic trauma occurs in people under 60 years of age worldwide. A study reports that there are accidents every minute and deaths every four minutes, mostly resulting from fractures. Consequently, they increase morbidity and burden healthcare resources to a greater extent.^4^

This study aimed to find out the prevalence of orthopaedic fractures among patients attending a tertiary care centre.

## METHODS

A descriptive cross-sectional study was conducted at the National Trauma Center, Kathmandu, Nepal, from 1 April to 30 September 2021. Data of patients admitted from 1 January to 30 December 2021 were collected. Data was retrieved from the hospital medical record section. The Institutional Review Committee of the National Academy of Medical Sciences, Mahabouddha, Kathmandu, Nepal, granted ethical approval (Reference number: 159/2080/81). The study included complete hospital data of patients who visited the hospital and were diagnosed with fractures. This study excluded patients who were admitted before or after the study timeframe and had incomplete study details. A convenience sampling method was used. The sample size was calculated using the following formula:


$$\begin{array}{ll} \text{n} & ={\text{Z}}^{2}\times \frac{\text{p}\times \text{q}}{{\text{e}}^{\text{2}}}\\ & =1.96^{2}\times \frac{0.50\times 0.50}{0.02^{2}}\\ & =2,401 \end{array}$$

Where,

n = required sample size
Z = 1.96 at 95% Confidence Interval (CI)
p = fracture prevalence
q = 1-p
e = margin of error, 5%

Hence, the minimum sample size required was 2,401.

A data collection sheet was used to document the demographic information (age and gender), vehicle details (vehicle type), and type of fractures sustained by each patient. Data anonymity was ensured by removing personal identifiers. Information regarding the fracture mode, including vehicle type, was provided by patients or their relatives. X-rays were taken to confirm the diagnosis of patients with fractures. After reviewing the images, the treating orthopaedic surgeons confirmed the fracture diagnosis.

Data were entered into Microsoft Excel 10 and analysed by using IBM SPSS Statistics version 17.0. A point estimate was calculated at a 95% CI.

## RESULTS

Among 7609 outpatients, 2518 (33.09%) (32.03-34.15, 95% CI) sustained fractures. The median age (interquartile range) of patients with fractures was 28 years (22-39). There were 1925 (76.45%) males and 593 (23.55%) females with fractures. There were 1018 (40.43%) patients aged 20-29 and 560 (22.24%) patients aged 20-29 and 30-39 (Table 1).

**Table 1:** Demographic of details patients with fracture (n= 2518).

Demographics		n (%)
	<10	69 (2.74)
	10-19	255 (10.13)
	20-29	1018 (40.43)
	30-39	560 (22.24)
Age group (years)	40-49	324 (12.87)
	50-59	168 (6.67)
	60-69	87 (3.46)
	70-79	29 (1.15)
	80-89	8 (0.32)
Gender	Male	1925 (76.45)
	Female	593 (23.55)

Out of 2518 fractures, 391 (15.53%) occurred in October, 344 (13.66%) in December, and 337 (13.38%) in November. There were 1012 (40.19%) fractures due to two-wheelers, while 424 (16.84%) due to four-wheelers. A total of 1082 (42.97%) fractures were not due to vehicular accidents (Table 2).

**Table 2:** Distribution of fractures due to vehicular accidents (n= 2518).

Vehicle	n (%)
Two-wheelers	1012 (40.19)
Four-wheelers	424 (16.84)
Others	1082 (42.97)

Among the total of 2518 fractures, there were 1387 (55.08%) soft tissue injuries, 626 (25.86%) upper extremity fractures, and 471 (18.71%) lower extremity fractures (Table 3).

**Table 3:** Types of fractures sustained among the patients (n= 2518).

Fractures		Overall Adult (n = 2239)	Age group			Gender	
			Children (n = 155)	Elderly (n = 124)	Female (n = 593)	Male (n = 1925)	
Upper extremity fracture	Facial injury	116 (4.61)	103 (4.60)	9 (5.81)	4 (3.23)	19 (3.20)	97 (5.04)
	Skull injury	198 (7.86)	164 (7.32)	12 (7.74)	22 (17.74)	38 (6.41)	160 (8.31)
	Clavical fracture	70 (2.78)	65 (2.90)	4 (2.58)	1 (0.81)	9 (1.52)	61 (3.17)
	Scapula fracture	9 (0.36)	8 (0.36)	1 (0.65)	0 (0)	1 (0.17)	8 (0.42)
	Shoulder dislocation	22 (0.87)	22 (0.98)	0 (0)	0 (0)	3 (0.51)	19 (0.99)
	Elbow fracture	16 (0.64)	16 (0.71)	0 (0)	0 (0)	3(0.51)	13 (0.68)
	Humerus fracture	48 (1.91)	40 (1.79)	6 (3.87)	2 (1.61)	11 (1.85)	37 (1.92)
	Radius fracture	68 (2.70)	57 (2.55)	6 (3.87)	5 (4.03)	18 (3.04)	50 (2.60)
	Ulna fracture	5 (0.20)	4 (0.18)	1 (0.65)	0 (0)	2 (0.34)	3 (0.16)
	Radius ulna fracture	22 (0.87)	20 (0.89)	2 (1.29)	0 (0)	6 (1.01)	16 (0.83)
	Scaphoid fracture	7 (0.28)	7 (0.31)	0 (0)	0 (0)	1 (0.17)	6 (0.31)
	Carpal and metacarpal fracture	39 (1.55)	37 (1.65)	2 (1.29)	0 (0)	1 (0.17)	38 (1.97)
	Other wrist fracture	6 (0.24)	5 (0.22)	1 (0.65)	0 (0)	2 (0.34)	4 (0.21)
Lower extremity fracture	Ribs fracture	3 (0.12)	3 (0.13)	0 (0)	0 (0)	1 (0.17)	2 (0.10)
	Spine injury	51 (2.03)	48 (2.14)	0 (0)	3 (2.42)	9 (1.52)	42 (2.18)
	Chest injury	33 (1.31)	31 (1.38)	1 (0.65)	1 (0.81)	5 (0.84)	28 (1.45)
	Hip fracture	9 (0.36)	8 (0.36)	0 (0)	1 (0.81)	2 (0.34)	7 (0.36)
	Pelvic fracture	15 (0.60)	12 (0.54)	1 (0.65)	2 (1.61)	7 (1.18)	8 (0.42)
	Knee dislocation	3 (0.12)	3 (0.13)	0 (0)	0 (0)	0 (0)	3 (0.16)
	Patella fracture	11 (0.44)	11 (0.49)	0 (0)	0 (0)	3 (0.51)	8 (0.42)
	Femur fracture	73 (2.90)	58 (2.59)	8 (5.16)	7 (5.65)	9 (1.52)	64 (3.32)
	Tibia fracture	40 (1.59)	55 (2.46)	5 (3.23)	7 (5.65)	10 (1.69)	57 (2.96)
	Fibula fracture	31 (1.23)	25 (1.12)	4 (2.58)	2 (1.61)	8 (1.35)	23 (1.19)
	Tibia fibula fracture	67 (2.66)	63 (2.81)	7 (4.52)	1 (0.81)	12 (2.02)	59 (3.06)
	Phalanx fracture	71 (2.82)	39 (1.74)	1 (0.65)	0 (0)	4 (0.67)	36 (1.87)
	Bimalleolar fracture	9 (0.36)	9 (0.40)	0 (0)	0 (0)	1 (0.17)	8 (0.42)
	Tarsal and metatarsal fracture	41 (1.63)	38 (1.70)	2 (1.29)	1 (0.81)	6 (1.01)	35 (1.82)
	Other ankle fracture	14 (0.56)	13 (0.58)	0 (0)	1 (0.81)	5 (0.84)	9 (0.47)
	Soft tissue injury	1,387 (55.08)	1244 (55.56)	80 (51.61)	63 (50.81)	390 (65.77)	997 (51.79)
	Abdominal trauma	20 (0.79)	18 (0.80)	1 (0.65)	1 (0.81)	2 (0.34)	18 (0.94)
	Crush injury	7 (0.28)	6 (0.27)	1 (0.65)	0 (0)	3 (0.51)	4 (0.21)
	Cut injury	7 (0.28)	7 (0.31)	0 (0)	0 (0)	2 (0.34)	5 (0.26)

## DISCUSSION

Hospital-visiting patients in this study had a fracture prevalence of 33.09%, which was 1.6-fold higher than in another study (20%).^5,6^ A similar incidence (33.93%) of fracture-associated hospital admissions has been reported in Nepal previously; however, another study found a varying prevalence between 2-15% of patients.^7^ It is likely that the differences in incidences are due to variations in the study population, from adults to children, and the geographic location of the hospitals. Because the patient population in our study centre tends to be traumatized, the prevalence is higher.

Patients' mean age in the present study was 28 years, and most were in the 20-29 (40.43%) and 30-39 (22.44%) age groups. However, it is worth noting that in multiple studies conducted in Nepal, the average age of patients with fractures ranged from 33.93 to 35 years.^6,7^ Similar to our findings, a study reported 25.47% of fractures in people between 30 and 45 years of age, followed by 15% in people 15 to 30 years of age.^8^ Similarly, the 10-39 age group represented over half of the traumas and fractures in another study.^5^ Adults in this study suffered more fractures due to accidents, specifically with two-wheelers, when they commuted to work or school or did not follow traffic laws. Men (76.45%) were more likely to suffer fractures in this study than women (23.55%), similar to the findings in other Nepalese studies.^5,8^ Our study found a 3.25:1 male-to-female ratio, while others found it varying from 1.82:1 to 1.52:1.^5,8^ As per a study from Nepal, the ratio of males to females in India (6:1) and Singapore (2.25:1) differed.^9,10^ A possible cause could be gender biases in work and educational opportunities and drunk-driving behaviours, particularly in developing countries, such as Nepal, which is dominated by men.

In this study, fractures were most commonly diagnosed in October (15.53%), December (13.67%), and November (13.38%). Another study reported higher prevalence in April, May, and June (38%), followed by July, August, and September (24%). Most importantly, this may be due to the different reporting of incidents based on seasons as cumulative incidences for months, as well as variations in social and cultural practices such as travelling during celebrations or holidays.^11^ The majority of orthopaedic events in this study involved soft tissue injuries (55.08%), followed by upper extremity fractures (25.86%) and lower extremity fractures (18.71%). Most fractures reported in several studies occurred on the upper limbs,^5,11^ while in a similar study conducted in England, lower limb fractures were most common.^12^ Fractures of the face (4.61%) and skull (786%) dominated upper extremity fractures, while femurs (2.90%) and the phalanx (2.82%) dominated lower extremity fractures. Two-wheeler riders who drive under the influence of alcohol and do not wear a helmet are more likely to suffer these fractures. As compared with another study (11.47%), our study found a lower prevalence of head injury (7.86%); yet the prevalence of chest injury (1.86%) was similar.^13^ Women sustained more soft tissue injuries in the present study (65%) than men (51%). Trauma sustained while cutting and dicing vegetables or falling while doing household chores could be to blame. In contrast, males in this study had higher rates of radius-ulna, carpal-metacarpal, and tibia-fibula fractures than females did. A significant part of this predilection towards males is due to the exposure males receive on two-wheelers while pursuing opportunities (education, work, refreshment) outside. Furthermore, patients aged <10 sustained the most soft tissue injuries (60.87%), followed by those aged 50-59 (58.33%) and 20-29 (56.58%). Injuries such as these are more common in children because of their playful nature, their predilection for running and jumping, which poses stresses on bones/joints, and their outdoor activities.

This study is a single-centre retrospective study so may not be generalizable to other settings. As a result, fracture prevalence may be underestimated or overestimated in the results.

## CONCLUSIONS

The prevalence of orthopaedic fractures among patients attending tertiary care centre is higher than other studies done in similar settings.
